# Prostatic carcinoma metastatic to frontal and cavernous sinuses: a case report^☆^

**DOI:** 10.1016/j.bjorl.2016.05.005

**Published:** 2016-06-25

**Authors:** Jérôme Rene Lechien, Jacques Doyen, Mohamad Khalife, Sven Saussez

**Affiliations:** a EpiCURA Hospital, RHMS Baudour, Department of Otolaryngology and Head and Neck Surgery, Baudour, Belgium; b University of Mons (UMONS), Faculty of Psychology, Research Institute for Language Sciences and Technology, Mons, Belgium; c University of Mons (UMONS), Faculty of Medicine, Research Institute for Health Sciences and Technology, Laboratory of Anatomy and Cell Biology, Mons, Belgium; d EpiCURA Hospital, RHMS Baudour, Department of Radiology, Baudour, Belgium

## Introduction

Primary malignancies of the nasal cavity and paranasal sinuses account for 0.3% of all head and neck cancers with a proportion of tumor in sphenoid and frontal sinuses estimated to 0.001%.^1^ Metastatic lesions in these anatomical sites are even rarer since they respectively concern 19% and 5% of all paranasal sinuses metastases.^2^ Paranasal metastases often come from kidney, lung, breast cancers and rarely from prostatic carcinomas since approximately 13 cases of prostatic metastasis in the nose and paranasal sinuses have been described in the literature.^3^, ^4^ Nowadays, it seems that only five cases of prostatic frontal metastasis were reported.^3^, ^5^, ^6^, ^7^ In this paper, we reported the case of a patient with a recurrence of a prostatic carcinoma manifested by concomitant metastases in both frontal and cavernous sinuses.

## Case report

**Figure 1:**
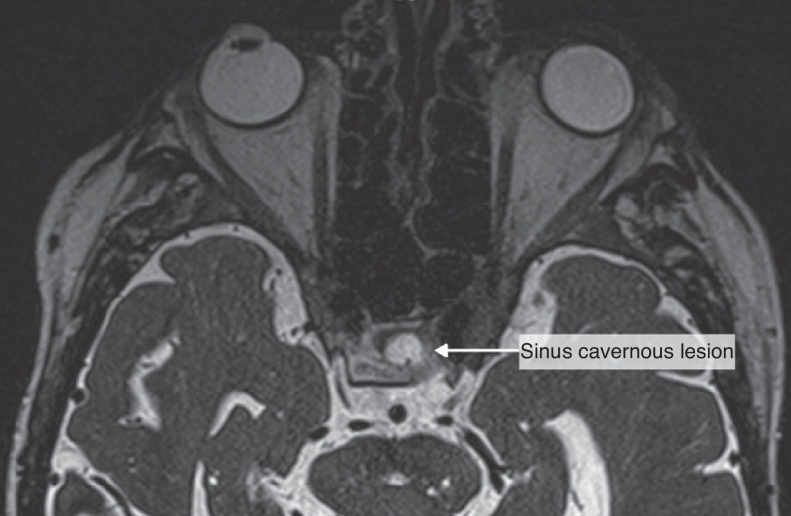
The sinus cavernous lesion (MRI, T2).

**Figure 2:**
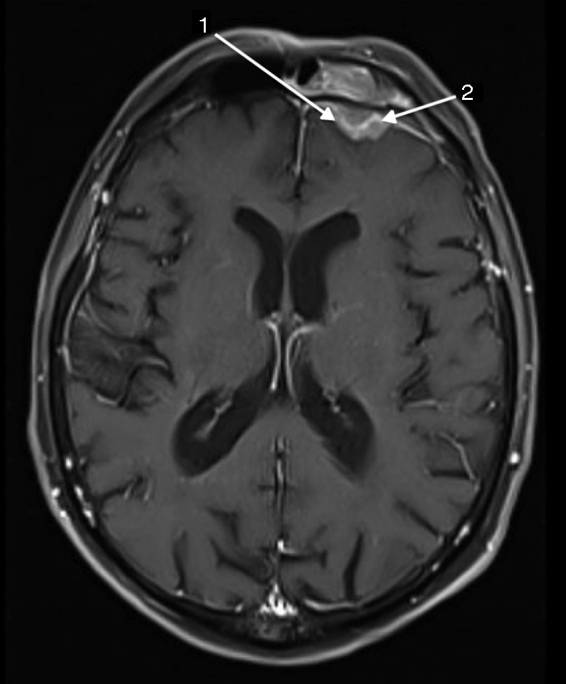
The sinus frontal lesion (MRI, T1). We observed *a* ≥ 2 mm dural thickening (1) and nodular dural enhancement (2) demonstrating the dural invasion.

**Figure 3:**
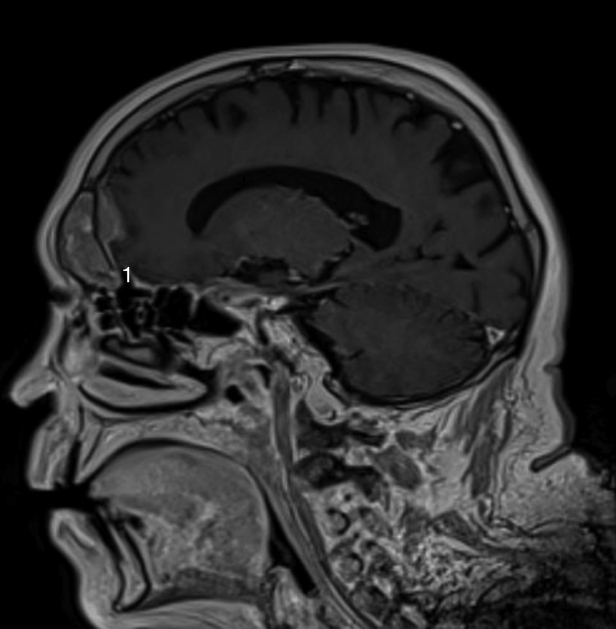
The extension of the sinus frontal metastasis with the loss of the bone hypointense zone showing a dural invasion (MRI, T1).

**Figure 4:**
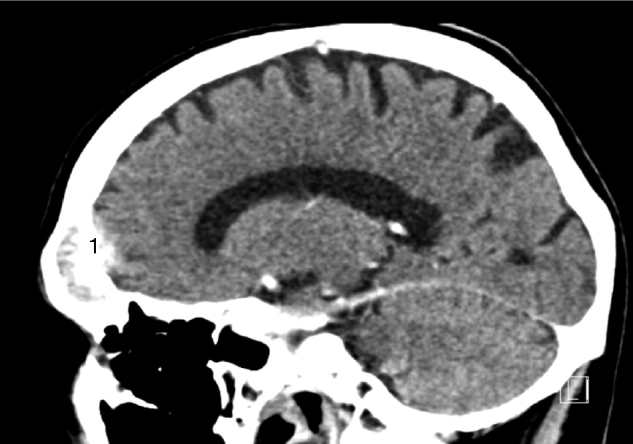
CT images showing a large frontal lesion with bone penetration (1).

A 67 year-old man was referred to the Department of Otolaryngology for the evaluation of a few days history of monocular diplopia, left facial and frontal pain. Patient did not report fever or continuous rhinorrheas. The general medical history revealed that the patient had previously been treated for a prostatic carcinoma by surgery, radiotherapy and hormonal therapy. The ENT clinical examination has just reported the presence of a divergent strabismus of the left eye and frontal pain increased on palpation. Concerning the nasofibroscopic examination, we did not found rhinorrhea, a nasal extension of the tumor and signs suggesting a complication of a rhinosinusitis. The frontal mass seemed not be complicated by a rhinosinusitis. A first physician suspected the diagnosis of a rhinosinusitis with a possible sinus cavernous thrombosis. Therefore, he prescribed a Magnetic Resonance Imaging (MRI) and a Computed Tomography (CT) that showed two focal lesions, the first in the left cavernous sinus and the second in the left frontal sinus (Figure 1, Figure 2). The lesion located in the left cavernous sinus measured 9.9 mm along the axis and infiltrated the left anterior clinoid process. The MRI also reported a T2 hypersignal soft tissue lesion appearing in the left frontal sinus with calcifications and with a gadolinium enhancement (34 mm along the axis). The signal abnormality of the medullary space of the left frontal bone, the bone destruction and erosion of the posterior wall of frontal sinus, and the extension to the frontal cortex suggested the possibility of a metastasis lesion (Figure 3, Figure 4). The whole body bone scintigraphy has been programmed and reported multiple metastases in cervical, dorsal, lumbar vertebrae and in pleural compartments. A palliative approach based on the continuation of the hormonal therapy (leuprorelin) was adopted given the advanced metastasis disease. Moreover, the palliative approach also consisted of a control of potential pain that might arise. The patient died a few months later without showing external mass on the forehead or additional neurological signs related to a potential frontal irritation.

## Discussion

The mechanism of prostatic metastases to the paranasal sinuses involves a spread cells that probably comes from the vertebral venous plexus.^5^ These metastases are characterized by similar complaints to the primary sinuses carcinoma i.e. epistaxis, facial pain, diplopia and other symptoms due to impairments of cranial nerve.^7^ Although it is described in most published cases, our patient did not report evident rhinoscopic and radiologic signs of rhinosinusitis even if it was the first diagnosis hypothesis. In the present case, the diplopia was secondary to another metastasis located in cavernous sinus leading to the palsy of the third cranial nerve. The frontal lesion had originated in the bone posterior wall of the frontal sinus and simultaneously extended to both frontal sinus and frontal cortex. This clinical presentation without external mass and with an extension to the cerebral anterior fossa was unusual for a frontal sinus metastasis. CT and MRI may specify the extension of the mass and whole body bone scintigraphy or PET/CT are crucial to exclude other metastases. Some studies suggested that malignant lesions could be diagnosed mainly by the existence of osteolytic destruction.^8^ Thus, the presence of a soft-tissue mass extending to the cerebral anterior fossa and the bone lysis seem to be common features of images described in paranasal malignancies.^8^ According to MacIntyre et al., *a* ≥ 2 ≥ mm dural thickening, the loss of hypointense zone (T1 signal with gadolinium) and nodular dural enhancement could be highly predictive for a dural invasion^9^ that is observed in the present case. By cons, it seems that there is no specific imaging sign to differentiate primary paranasal cancer from metastasis.^4^ These specific MRI findings that have a high predictive value on the malignancy diagnosis, are described in our case. Except in a single metastasis case, the role of the functional endoscopic sinus surgery is limited to the diagnosis biopsy and the control of the symptoms by a reduction of the tumor compression. Given the advanced metastasis disease of our patient, the biopsy was not considered to be appropriate. The treatment of frontal sinus metastasis depends of the extension of the disease and, when there is an isolated metastasis, it has not been established in view of the paucity of retrospective case studies. It seems that the utilization of chemotherapy and radiotherapy report mixed results and remain controversial.^10^ In this case, we based the treatment on a palliative hormonal therapy owing to the hormonal characteristics of the primary tumor and the general metastatic disease. Although these lesions are rare, it seems that the metastatic disease in paranasal sinuses is characterized by a poor prognosis leading to the death within the next months.^5^, ^6^

## Conclusion

Concomitant metastases of the frontal and cavernous sinuses are a rare condition that can lead to a misdiagnosis of rhinosinusitis. In the context of a history of cancer, even if it remains exceptional, an imaging must be made to precise the etiology of the symptoms. In the case of frontal malignancy, dural thickening and nodular enhancement combined with the loss of hypointense zone could be highly predictive for a dural invasion implying a poor prognosis.

## Conflicts of interest

The authors declare no conflicts of interest.
